# Reproducibility and uptake time dependency of volume-based parameters on FDG-PET for lung cancer

**DOI:** 10.1186/s12885-016-2624-3

**Published:** 2016-08-02

**Authors:** Tomoka Kitao, Kenji Hirata, Katsumi Shima, Takashi Hayashi, Mitsunori Sekizawa, Toshiki Takei, Wataru Ichimura, Masao Harada, Keishi Kondo, Nagara Tamaki

**Affiliations:** 1 Radiology Department, National Hospital Organization, Hokkaido Cancer Center, 2-3-54, Kikusui-4, Shiroishi-Ku, Sapporo, 003-0804 Japan; 2 Department of Nuclear Medicine, Graduate School of Medicine, Hokkaido University, Kita 15, Nishi 7, Kita-Ku, Sapporo, Hokkaido 060-8638 Japan; 3 Department of Diagnostic Radiology, Hokkaido Cancer Center, Sapporo, Japan; 4 Department of Respiratory Medicine, Hokkaido Cancer Center, Sapporo, Japan; 5 Department of Thoracic Surgery, Hokkaido Cancer Center, Sapporo, Japan

**Keywords:** Lung cancer, Reproducibility, Metabolic tumor volume, Total lesion glycolysis, FDG-PET

## Abstract

**Background:**

Volume-based parameters, such as metabolic tumor volume (MTV) and total lesion glycolysis (TLG), on F-18 fluorodeoxyglucose (FDG) positron emission tomography (PET) are useful for predicting treatment response in nonsmall cell lung cancer (NSCLC). We aimed to examine intra- and inter-operator reproducibility to measure the MTV and TLG, and to estimate their dependency on the uptake time.

**Methods:**

Fifty NSCLC patients underwent preoperative FDG-PET. After an injection of FDG, the whole body was scanned twice: at the early phase (61.4 ± 2.8 min) and delayed phase (117.7 ± 1.6 min). Two operators independently defined the tumor boundary using three different delineation methods: (1) the absolute SUV threshold method (MTV_p_ and TLG_p_; *p* = 2.0, 2.5, 3.0, 3.5), (2) the fixed% SUVmax threshold method (MTV_q%_ and TLG_q%_; q = 35, 40, 45), and (3) the adaptive region-growing method (MTV_ARG_ and TLG_ARG_). Parameters were compared between operators and between phases.

**Results:**

Both the intra- and inter-operator reproducibility were high for all parameters using any method (intra-class correlation > 0.99 each). MTV_3.0_ and MTV_3.5_ resulted in a significant increase from the early to delayed phase (*P* < 0.05 for both), whereas MTV_2.0_ and MTV_2.5_ neither increased nor decreased (*P* = n.s.). All of the MTV_q%_ values significantly decreased over time (*P* < 0.01), whereas MTV_ARG_ and TLG with any delineation method increased significantly (*P* < 0.05).

**Conclusions:**

High reproducibility of MTV and TLG was obtained by all of the methods used. MTV_2.0_ and MTV_2.5_ were the least sensitive to uptake time, and may be good alternatives when we compare images acquired with different uptake times, although applying constant uptake time is important for volume measurement.

## Background

Positron emission tomography (PET) using F-18 fluorodeoxyglucose (FDG) has been an essential diagnostic tool in oncology [1–3]. FDG-PET generates functional images that contribute to clinical diagnoses and treatment planning complementarily with anatomical modalities such as computed tomography (CT) and magnetic resonance imaging (MRI). PET is also characterized by high quantitative performance [4–6]. In most clinical settings, FDG-PET images were assessed semi-quantitatively using the standardized uptake value (SUV), which commonly represents the radioactivity concentration per unit volume of tissue normalized to the injected dosage and body weight [7]. The maximum of the SUV (SUVmax) within the tumor has been used most frequently to express the intensity of FDG uptake in the tumor because of its simplicity and high reproducibility [8–12]. However, the SUVmax has several problems. Because the SUVmax represents just a single voxel (normally < 0.1 ml) and not the entire tumor metabolism, it is sensitive to statistical noise of the image [13]. In recent years, the use of the SUVpeak has been preferred [13]. The definition of SUVpeak remains to be standardized, but usually calculated by averaging SUV within a 1-ml sphere (12 mm in diameter) around the voxel showing highest intensity voxel. The SUVpeak is less sensitive to image noise but suffers from the same problem as SUVmax still reflects a small part of the tumor [14, 15].

In this context, the metabolic tumor volume (MTV) and total lesion glycolysis (TLG) has been recently used as indices of the whole tumor FDG uptake. The MTV is defined as the volume of tumor determined on an FDG-PET image using a certain threshold. Once MTV is determined, the SUVmean can be defined as the averaged SUV within MTV. TLG is the product of the MTV and the SUVmean. These indicators reflect the activity of the glucose metabolism in the entire tumor. The clinical usefulness of these indicators (e.g., prognosis and treatment response) has been demonstrated in many cancers such as lung [16, 17], head-and-neck [18–20], and gynecological cancer [21, 22].

Calculating the MTV and TLG requires tumor contouring on the PET image. Many methods have been reported to determine the contour [23–31], and among them, manual contouring, the absolute SUV threshold method, and relative SUV threshold methods have been used widely. With the manual contouring method, the tumor boundary is determined based on an operator’s visual inspection. This operator-dependent method suffers from reproducibility and is affected by the window level and color scale. It also takes a long time to apply this manual operation to all of the images containing tumors. Other methods have thus been developed to reduce the effects from display conditions or operators.

There is no doubt that the SUVmax has high intra- and inter-operator reproducibility, but the reproducibility of MTV and TLG still needs to be assessed. In the present study, we examined intra-operator reproducibility (i.e., the same operator analyzes the same image twice) and inter-operator reproducibility (i.e., two operators analyze the same image independently). In addition, considering possible effects of uptake time after the FDG administration on the MTV and TLG, we acquired PET images twice after a single injection (at 60 and 120 min), and we compared the MTV and TLG between these images. We applied different delineation methods that are widely used. Thus, in this study, we aimed to evaluate (1) intra-operator reproducibility, (2) inter-operator reproducibility, and (3) the effect of uptake time differences on volume-based parameters.

## Methods

### Study subjects

All procedures performed in studies involving human participants were in accordance with the ethical standards of the institutional and/or national research committee and with the 1964 Helsinki declaration and its later amendments or comparable ethical standards. The institutional ethics committee of Hokkaido Cancer Center approved this retrospective study. Informed consent was waived from individual participants in the retrospective study according to the committee. Patient records/information was anonymized and de-identified prior to analysis. From our hospital information system, we found a total of 52 patients who underwent FDG-PET for an examination of lung nodules before treatment at the National Hospital Organization Hokkaido Cancer Center between December 2010 and March 2012. One patient was suspected of having metastatic lung tumor from breast cancer, and another patient did not complete the scanning because of severe pain. Thus, we included 50 patients (27 males; age, 70.2 ± 10.1 years old) whose lung nodules were visualized by FDG-PET and whose nodule(s) were pathologically confirmed as non-small cell lung cancer (NSCLC). The patient characteristics are shown in Table 1. Briefly, body weight was 56.0 ± 9.0 kg (range 39–87 kg); tumor existed in the upper lobe (*N* = 30), the middle lobe (*N* = 4), or the lower lobe (*N* = 16) of the lung; pathological diagnosis was adenocarcinoma (*N* = 28), squamous cell carcinoma (*N* = 14), or others (*N* = 8); cancer stage was IA (*N* = 12), IB (*N* = 6), IIA (*N* = 10), IIB (*N* = 3), IIIA (*N* = 8), IIIB (*N* = 5) or IV (*N* = 6) based on the American Joint Committee on Cancer (AJCC) TNM system.

**Table 1:** Patient characteristics

Case	Age range (y)	Weight (kg)	Dosage (MBq)	Dosage/Weight (MBq/kg)	Tumor location	Pathology^a^	TNM classification	Stage
1	80–89	43.3	142	3.3	L S3	Ade	pT1aN0M0	IA
2	70–79	56.5	166	2.9	R S6	SCC	cT3N2M0	IIIA
3	80–89	50.0	239	4.8	L S1 + 2	Ade	cT2aN0M0	IB
4	60–69	46.3	162	3.5	L S8	SCC	pT2aN0M0	IB
5	80–89	52.6	283	5.4	R S7	Ade	pT2aN2M0	IB
6	60–69	63.0	164	2.6	R S6	SCC	pT1aN0M0	IA
7	70–79	48.6	146	3.0	R S3	Ade	pT1bN0M0	IA
8	70–79	46.5	144	3.1	R S2	Ade	pT2aN1M0	IIA
9	70–79	72.4	292	4.0	R S5	SCC	pT1aN0M0	IA
10	70–79	47.4	242	5.1	R S1	Ade	cT4N2M0	IIIB
11	30–39	40.7	147	3.6	R S8	MEDC	pT1bN0M0	IA
12	70–79	45.9	294	6.4	L S9	Ade	pT1bN0M0	IA
13	50–59	50.0	239	4.8	R S1	Ade	pT1aN0M0	IA
14	60–69	65.7	276	4.2	L S1 + 2	SCC	cT4N3M1b	IV
15	50–59	60.7	294	4.8	L S5	Ade	pT2aN1M1b	IV
16	60–69	52.1	243	4.7	R S2	SCC	cT4N3M0	IIIB
17	80–89	56.2	236	4.2	R S2	Ade	pT1aN0M0	IA
18	70–79	57.4	145	2.5	R S7	LCNEC	pT2aN2M0	IB
19	70–79	78.4	242	3.1	L S1 + 2	SCC	pT1bN0M0	IA
20	50–59	60.6	242	4.0	R S1	Ade	pT1bN2M0	IIIA
21	60–69	67.2	243	3.6	L S3	Ade	pT3N1M0	IIIA
22	80–89	48.5	240	5.0	R S10	Ade	pT2bN0M0	IIA
23	60–69	64.7	243	3.8	L S10	SCC	pT3N0M0	IIB
24	70–79	51.4	242	4.7	L S9	Ade	pT2bN0M0	IIA
25	60–69	54.8	292	5.3	R S3	Ade	pT2aN0M0	IIA
26	60–69	45.9	224	4.9	L S1 + 2	Ade	pT4N0M0	IIIA
27	80–89	62.7	242	3.9	L S1 + 2	SCC	cT3N0M0	IIB
28	70–79	55.4	146	2.6	R S4	Ade	cT2bN1M1b	IV
29	60–69	59.4	239	4.0	R S2	Ade	pT2bN0M0	IIA
30	70–79	53.0	145	2.7	L S10	PC	pT2aN1M0	IIB
31	70–79	55.3	237	4.3	R S1	PC	pT1aN0M0	IA
32	70–79	50.7	146	2.9	L S4	Ade	pT1aN3M0	IIIB
33	60–69	68.8	274	4.0	R S5	Ade	cT2aN2M1	IV
34	60–69	57.2	245	4.3	R S1	SCC	cT3N2M0	IIIA
35	70–79	45.6	242	5.3	R S1	Ade	pT1aN0M0	IA
36	80–89	54.6	274	5.0	L S1 + 2	Ade	pT3N1M0	IIIA
37	70–79	46.6	146	3.1	R S3	Ade	pT1aN2M0	IIIA
38	80–89	51.4	292	5.7	R S10	LCNEC	pT2aN0M0	IIA
39	80–89	74.8	273	3.6	R S4	Ade	pT2aN0M0	IIA
40	60–69	51.9	166	3.2	R S3	Ade	cT2aN0M1b	IV
41	70–79	40.8	164	4.0	R S3	Ade	pT2aN0M0	IB
42	50–59	45.2	166	3.7	L S3	SCC	cT4N2M0	IIIB
43	50–59	61.4	266	4.3	R S7	PC	pT2aN2M1	IV
44	50–59	72.2	290	4.0	L S10	SCC	cT2bN3M0	IIIB
45	60–69	62.4	166	2.7	R S1	LCNEC	pT2aN0M0	IIA
46	60–69	54.3	274	5.1	R S8	Ade	pT1aN0M0	IA
47	70–79	59.4	164	2.8	R S3	SCC	pT2aN0M0	IB
48	60–69	65.6	239	3.6	L S3	Ade	pT2aN1M0	IIA
49	70–79	61.9	292	4.7	R S3	SCC	pT1bN2M0	IIIA
50	60–69	60.9	290	4.8	R S6	ASCC	pT1aN1M0	IIA

^a^ *Ade* adenocarcinoma, *SCC* squamous cell carcinoma, *ASCC* adenosquamous carcinoma, *PC* pleomorphic carcinoma, *MEDC* mucoepidemoid carcinoma, *LCNEC* large cell neuroendocrine carcinoma

### Image acquisition and reconstruction

All of the clinical FDG-PET studies were performed with an Eminence SET-3000G PET scanner (Shimadzu, Kyoto, Japan). All of the patients fasted for at least 6 h before the injection of FDG (224 ± 54 MBq, range 142–294 MBq; 4.0 ± 0.9 MBq/kg, range 2.5–6.4 MBq/kg). The blood glucose level was 100 ± 19 mg/dl. The images were scanned twice for each study: early scanning at 61.4 ± 2.8 min (range 58–67 min) and delayed scanning at 117.7 ± 1.6 min (range 114–121 min). The transaxial field of view was 512 mm in diameter. Three-dimensional emission scanning was performed in a continuous bed-movement manner (0.8–0.9 mm/s). Transmission scanning was performed with a ^137^Cs external source to correct for attenuation.

Images were reconstructed with a block-iterative algorithm named ‘dynamic row-action maximum likelihood algorithm (DRAMA),’ modified from the row-action maximum likelihood algorithm (RAMLA) [32]. The iteration and filter cycle values for DRAMA were 1 and 128, respectively. The reconstructed image had a spatial resolution of 8.4 mm full-width at half-maximum and a matrix size of 128 × 128 with the voxel size 4.0 × 4.0 × 2.0 mm. A smoothing filter was not applied.

### Image processing

**Fig. 1:**
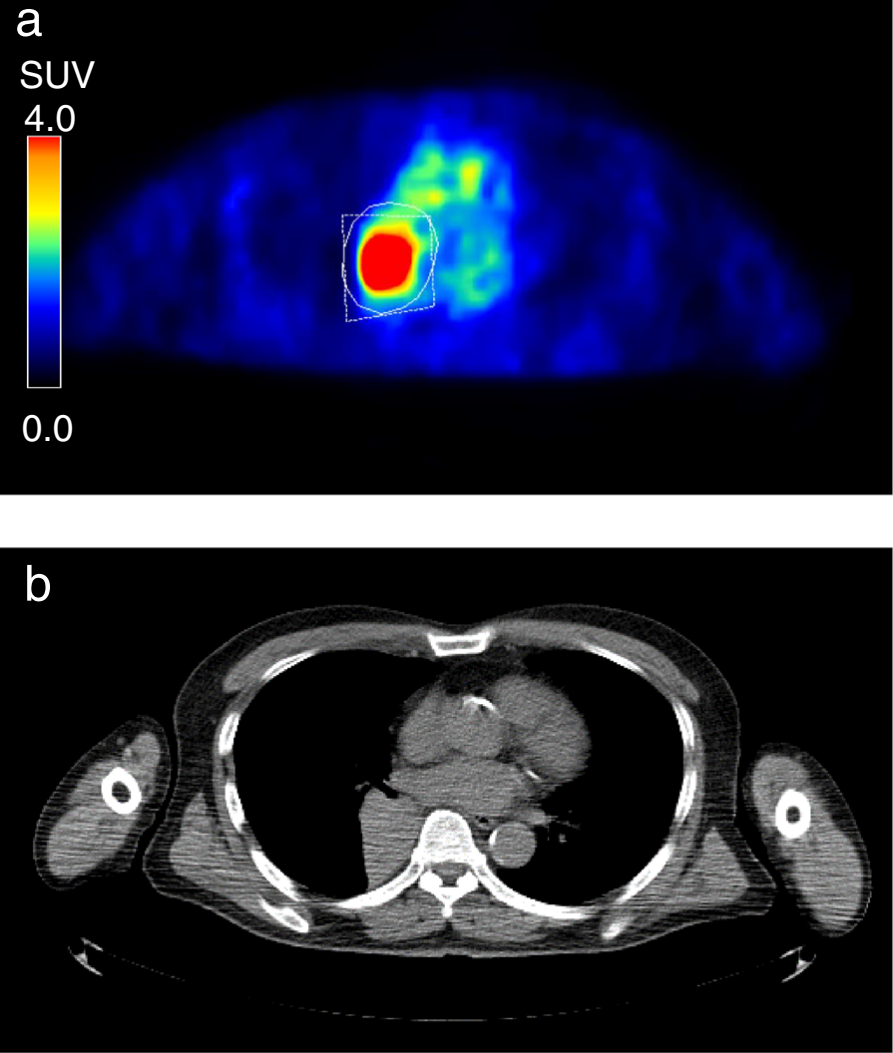
**a** Regions of interest defined by Operator-1 (solid line) and Operator-2 (dashed line). **b** Corresponding CT slice

A total of 100 FDG-PET datasets (two datasets, i.e., early and delayed images, from 50 patients) were processed to delineate the tumor by two operators (Fig. 1). Operator-1 (T.K.) is an experienced radiologic technologist of nuclear medicine, and Operator-2 (K.H.) is an experienced nuclear medicine physician. Both Operator-1 and Operator-2 independently defined the tumor boundary two times with an interval of 30 days or longer (i.e., a total of 4-time measurements). Operator-2 defined the tumor boundary once without viewing the results reported by Operator-1, and vice versa. Hereinafter, we use these three abbreviations: Op_1_Ob_1_ representing the first observation from operator-1, Op_1_Ob_2_ representing the second observation from operator-1, Op_2_Ob_1_ representing the first observation from Operator-2, and Op_2_Ob_2_ representing the second observation from Operator-2. The volume-of-interest (VOI) was defined by manually drawing polygonal regions of interest (ROIs) to enclose the entire tumor with enough margins on every slice where the tumor was seen. During the ROI definition, the PET images were displayed using a rainbow color bar with a fixed window level of SUV 0–4. Physiological uptake was carefully avoided. Neither lymph nodes nor distant metastatic lesions were investigated in this study. All of the ROIs were combined to generate a three-dimensional VOI.

In this study, we used the following three delineation methods. (1) The absolute SUV threshold method, which is a procedure of defining the area of the tumor as a region with a certain value higher than predetermined threshold, such as an SUV of 2.5 or 3.0. (2) The fixed% SUVmax threshold method, which is a procedure for defining the area of the tumor as a region with a higher SUV than a certain percentage of the SUVmax within the tumor (40–50 %, commonly). (3) The adaptive region-growing method (ARG), which is a relatively new method [26]. The ARG is essentially a region-growing method that examines neighboring voxels of the current region and determines whether the neighbor voxels should be added to the in-tumor region. If {a neighbor voxel} ≥ {mean of current region} × {arbitrary threshold}, the voxel is added to the region. There is a sharp volume increase point when the threshold (%) varies from 100 to 0 %, and the tumor region is determined by this border point. With this method, the area of the tumor can be extracted automatically by the setting of the highest voxel in the tumor. Because the ARG method uses a new procedure, there are still few studies using this method.

The tumor volume was automatically determined within the VOI using different methods: MTV_p_, MTV_q%_, and MTV_ARG_. MTV_p_ is the MTV determined using the absolute SUV threshold method, where *p* = 2.0, 2.5, 3.0, or 3.5. MTV_q%_ is the MTV determined using the fixed% SUVmax threshold method, where q = 35, 40, or 45 %. Values of p and q were chosen based on their frequency of appearance in literature [13]. MTV_ARG_ is the MTV determined using ARG method.

TLG was defined as the product of the corresponding MTV and SUVmean values within the tumor boundary. The SUVmax was also recorded, which represented the voxel showing the highest SUV in the VOI. The SUV was calculated as [tissue radioactivity concentration (Bq/ml)] × [body weight (g)] /[injected radioactivity (Bq)].

For all the image analysis including manual ROI drawing, mathematical delineation, and parameter calculation, we used an in-house software package, composed with Visual Studio 2010 (Microsoft Corporation, Redmond, Washington, USA) and C# language.

### Statistical analysis

Values are expressed as the mean ± SD. The free statistical package R version 3.2.5 (R Project, http://cran.r-project.org) was used for all statistical analyses. A paired *t*-test was used if the values could be considered paired. The method of Holm was used to adjust the *P*-values for multiple comparisons. The intra-class correlation (ICC) was used to evaluate intra- and inter-operator reproducibility [33]. Intra-operator reproducibility was estimated by 2 combinations: 1) Op_1_Ob_1_ vs. Op_1_Ob_2_, and 2) Op_2_Ob_1_ vs. Op_2_Ob_2_. Inter-operator reproducibility can be estimated by 4 combinations: 1) Op_1_Ob_1_ vs. Op_2_Ob_1_, 2) Op_1_Ob_1_ vs. Op_2_Ob_2_, 3) Op_1_Ob_2_ vs. Op_2_Ob_1_, and 4) Op_1_Ob_2_ vs. Op_2_Ob_2_. *P*-values <0.05 were considered as significant.

## Results

### Reproducibility

Both intra- and inter-operator reproducibility were extremely high at the early phase (Table 2) and the delayed phase (Table 3). The ICC between the first versus second measurement by Operator-1 or Operator-2 was > 0.99 for any parameters. Similarly, the ICC between Operator-1 versus Operator-2 was > 0.99 for any parameters. Among the parameters, no difference was observed in SUVmax, MTV_ARG_ or TLG_ARG_ in any case (i.e., perfect match). Comparisons between methods revealed that most of the MTV_q%_ values were lower than those of MTV_p_ or MTV_ARG_.

**Table 2:** Intra- and inter-operator reproducibility of PET parameters at the early phase

	Op1Ob1	Op1Ob2	Op2Ob1	Op2Ob2	ICC intra-operator reproducibility		ICC inter-operator reproducibility			
	n = 50				Op1Ob1 vs. Op1Ob2	Op2Op1 vs. Op2Op2	Op1Ob1 vs. Op2Ob1	Op1Ob1 vs. Op2Ob2	Op1Ob2 vs. Op2Ob1	Op1Ob2 vs. Op2Ob2
SUV_max_	9.1 ± 4.9	9.1 ± 4.9	9.1 ± 4.9	9.1 ± 4.9	1	1	1	1	1	1
MTV_2.0_	55.0 ± 100.6	54.9 ± 102.5	52.8 ± 96.6	52.8 ± 96.4	>0.999	>0.999	0.997	0.997	0.997	0.997
MTV_2.5_	39.4 ± 72.1	39.3 ± 72.5	39.1 ± 70.7	38.9 ± 70.4	>0.999	>0.999	>0.999	>0.999	>0.999	0.999
MTV_3.0_	29.7 ± 51.7	29.7 ± 51.6	29.8 ± 51.3	29.7 ± 51.1	>0.999	>0.999	>0.999	>0.999	>0.999	>0.999
MTV_3.5_	23.4 ± 38.2	23.4 ± 38.1	23.5 ± 38.0	23.4 ± 37.8	>0.999	>0.999	>0.999	>0.999	>0.999	>0.999
MTV_35%_	25.0 ± 31.9	24.3 ± 31.7	23.7 ± 31.5	23.7 ± 31.5	0.998	>0.999	0.997	0.992	0.997	0.997
MTV_40%_	19.2 ± 22.9	18.9 ± 22.8	18.6 ± 22.7	18.6 ± 22.7	0.998	>0.999	0.998	0.996	0.998	0.998
MTV_45%_	15.0 ± 17.1	14.9 ± 17.1	14.8 ± 16.9	14.8 ± 16.9	>0.999	1	>0.999	0.999	>0.999	>0.999
MTV_ARG_	43.0 ± 62.1	43.0 ± 62.1	43.0 ± 62.1	43.0 ± 62.1	1	1	1	1	1	1
TLG_2.0_	225.2 ± 365.2	224.9 ± 369.0	220.4 ± 354.5	220.0 ± 353.5	>0.999	>0.999	0.999	0.999	0.999	0.999
TLG_2.5_	190.4 ± 304.6	190.1 ± 305.5	189.8 ± 300.1	189.2 ± 298.9	>0.999	>0.999	>0.999	>0.999	>0.999	>0.999
TLG_3.0_	164.0 ± 253.7	163.7 ± 253.4	164.4 ± 251.8	163.8 ± 250.7	>0.999	>0.999	>0.999	>0.999	>0.999	>0.999
TLG_3.5_	143.6 ± 215.0.	143.4 ± 214.7	144.1 ± 213.7	143.6 ± 212.9	>0.999	>0.999	>0.999	>0.999	>0.999	>0.999
TLG_35%_	132.7 ± 179.5	131.8 ± 179.7	131.4 ± 179.2	131.3 ± 179.1	>0.999	>0.999	>0.999	>0.999	>0.999	>0.999
TLG_40%_	112.8 ± 148.3	112.3 ± 148.3	112.2 ± 147.7	112.1 ± 147.7	>0.999	>0.999	>0.999	>0.999	>0.999	>0.999
TLG_45%_	96.2 ± 124.7	96.0 ± 124.7	96.0 ± 123.9	96.0 ± 123.9	>0.999	1	>0.999	>0.999	>0.999	>0.999
TLG_ARG_	196.4 ± 290.0	196.4 ± 290.0	196.4 ± 290.0	196.4 ± 290.0	1	1	1	1	1	1

*ICC* intra-class correlation, *Op1Ob1* Operator-1's first observation, *Op1Ob2* Operator-1's second observation, *Op2Ob1* Operator-2's first observation, *Op2Ob2* Operator-2's second observation

**Table 3:** Intra- and inter-operator reproducibility of PET parameters at the delayed phase

	Op1Ob1	Op1Ob2	Op2Ob1	Op2Ob2	ICC intra-operator reproducibility		ICC inter-operator reproducibility			
	n = 50				Op1Ob1 vs. Op1Ob2	Op2Op1 vs. Op2Op2	Op1Ob1 vs. Op2Ob1	Op1Ob1 vs. Op2Ob2	Op1Ob2 vs. Op2Ob1	Op1Ob2 vs. Op2Ob2
SUV_max_	11.1 ± 6.0	11.1 ± 6.0	11.1 ± 6.0	11.1 ± 6.0	1	1	1	1	1	1
MTV_2.0_	56.2 ± 107.2	56.2 ± 107.9	54.1 ± 101.0	54.7 ± 101.8	>0.999	>0.999	0.996	0.995	0.993	0.994
MTV_2.5_	41.9 ± 79.6	41.8 ± 79.3	41.6 ± 77.6	41.9 ± 78.0	>0.999	>0.999	0.999	0.998	0.997	0.997
MTV_3.0_	33.1 ± 60.9	33.0 ± 60.3	33.5 ± 60.6	33.3 ± 60.3	>0.999	>0.999	>0.999	0.998	0.998	0.998
MTV_3.5_	27.3 ± 48.2	27.2 ± 47.7	27.5 ± 47.8	27.7 ± 48.1	>0.999	>0.999	>0.999	0.998	0.998	0.998
MTV_35%_	21.0 ± 29.0	20.9 ± 28.7	20.5 ± 28.7	20.6 ± 28.8	>0.999	>0.999	0.998	0.997	0.996	0.997
MTV_40%_	16.6 ± 21.2	16.5 ± 21.0	16.4 ± 21.0	16.5 ± 21.1	>0.999	>0.999	0.999	0.999	0.998	0.999
MTV_45%_	13.2 ± 15.7	13.1 ± 15.5	13.1 ± 15.5	13.1 ± 15.6	>0.999	>0.999	>0.999	>0.999	>0.999	>0.999
MTV_ARG_	50.4 ± 76.2	50.4 ± 76.2	50.4 ± 76.2	50.4 ± 76.2	1	1	1	1	1	1
TLG_2.0_	259.1 ± 431.3	258.6 ± 430.8	254.4 ± 414.8	256.8 ± 418.8	>0.999	>0.999	0.998	0.997	0.996	0.997
TLG_2.5_	227.2 ± 372.8	226.4 ± 370.5	226.4 ± 365.4	228.2 ± 368.4	>0.999	>0.999	>0.999	0.998	0.998	0.998
TLG_3.0_	203.4 ± 325.8	202.6 ± 322.9	205.4 ± 324.8	203.8 ± 322.0	>0.999	>0.999	>0.999	0.999	0.998	0.998
TLG_3.5_	184.5 ± 288.8	183.8 ± 286.3	184.9 ± 285.5	186.5 ± 288.3	>0.999	>0.999	>0.999	0.999	0.998	0.999
TLG_35%_	145.2 ± 206.5	144.5 ± 204.8	144.3 ± 204.7	145.4 ± 206.3	>0.999	>0.999	>0.999	>0.999	0.999	0.999
TLG_40%_	125.8 ± 173.3	125.2 ± 171.9	125.0 ± 171.2	126.0 ± 172.6	>0.999	>0.999	>0.999	>0.999	0.999	>0.999
TLG_45%_	108.1 ± 145.3	107.6 ± 144.1	107.3 ± 143.2	108.1 ± 144.3	>0.999	>0.999	>0.999	>0.999	>0.999	>0.999
TLG_ARG_	249.4 ± 383.5	249.4 ± 383.5	249.4 ± 383.5	249.4 ± 383.5	1	1	1	1	1	1

*ICC* intra-class correlation, *Op1Ob1* Operator-1's first observation, *Op1Ob2* Operator-1's second observation, *Op2Ob1* Operator-2's first observation, *Op2Ob2* Operator-2's second observation

### Parameter changes from the early phase to the delayed phase

**Fig. 2:**
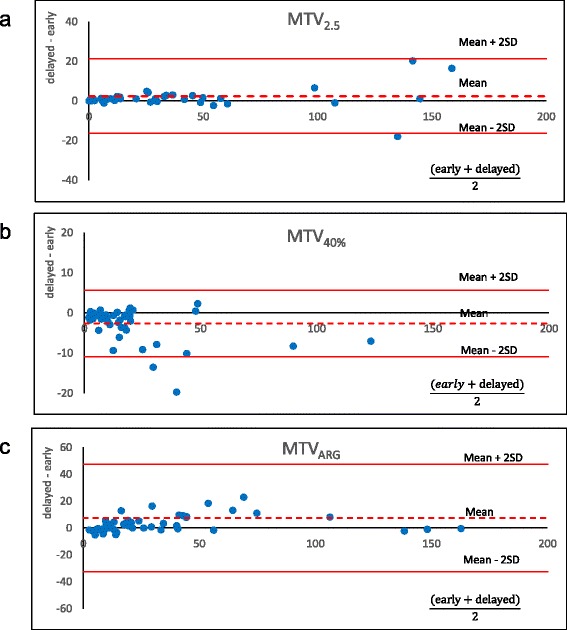
Bland-Altman plots showing the parameter changes between the early phase and delayed phase of the MTV, which is a general threshold value. MTV_2.5_ had few parameter changes (**a**), MTV_40%_ decreased (**b**), and MTV_ARG_ increased (**c**) from the early phase to the delayed phase

**Fig. 3:**
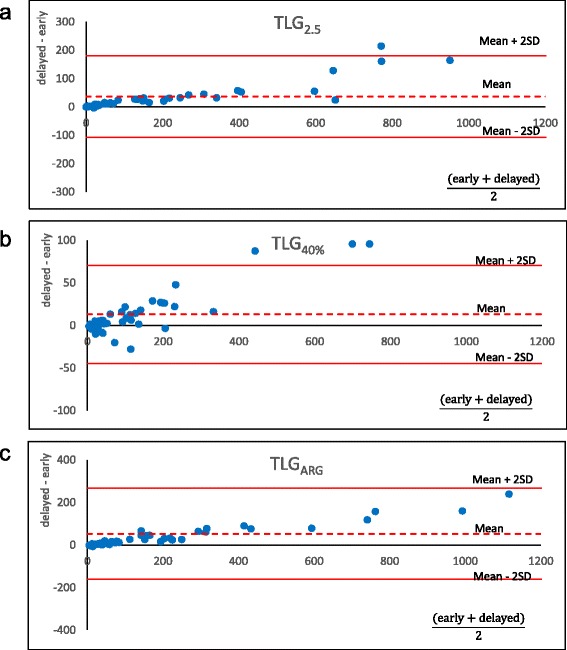
Bland-Altman plots showing the parameter changes between the early phase and the delayed phase of TLG, which is general threshold value. Unlike Fig. 2, TLG obtained by any delineation method was increased at the delayed phase (**a**-**c**)

Parameter changes from early to delayed phases are summarized in Table 4. The SUVmax increased in 49 of the 50 (98 %) cases at the delayed phase compared to the early phase (early, 9.1 ± 4.9; delayed, 11.1 ± 6.0; *P* < 0.0001). The MTV changes depended on the delineation methods. Among them, the MTV_2.0_ and MTV_2.5_ neither increased nor decreased from the early phase to the delayed phase with the averaged delayed-to-early ratios of 1.02 and 1.06, respectively (*P* = nonsignificant for both). The use of a higher threshold (i.e., MTV_3.0_ and MTV_3.5_) led to a significant increase from the early to the delayed phase (*P* < 0.05 for both). All of the MTV_q%_ values (i.e., MTV_35%_, MTV_40%_, and MTV_45%_) significantly decreased (*P* < 0.001), whereas the MTV_ARG_ values significantly increased (*P* < 0.05) (Fig. 2). In contrast, the TLG obtained by any of the delineation methods was significantly increased at the delayed phase (Fig. 3).

**Table 4:** The parameter changes from the early phase to the delayed phase

	Early Phase (E)	Delayed Phase (D)	D/E (mean)	*P*-value
	*n* = 50			
SUV_max_	9.1 ± 4.9	11.1 ± 6.0	1.22	<0.0001
MTV_2.0_	55.0 ± 100.6	56.2 ± 107.2	1.02	NS
MTV_2.5_	39.4 ± 72.1	41.9 ± 79.6	1.06	NS
MTV_3.0_	29.7 ± 51.7	33.1 ± 60.9	1.11	<0.05
MTV_3.5_	23.4 ± 38.2	27.3 ± 48.2	1.17	<0.05
MTV_35%_	25.0 ± 31.9	21.0 ± 29.0	0.84	<0.001
MTV_40%_	19.2 ± 22.9	16.6 ± 21.2	0.86	<0.001
MTV_45%_	15.0 ± 17.1	13.2 ± 15.7	0.88	<0.001
MTV_ARG_	43.0 ± 62.1	50.4 ± 76.2	1.17	<0.05
TLG_2.0_	225.2 ± 365.2	259.1 ± 431.3	1.15	<0.01
TLG_2.5_	190.4 ± 304.6	227.2 ± 372.8	1.19	<0.001
TLG_3.0_	164.0 ± 253.7	203.4 ± 325.8	1.24	<0.001
TLG_3.5_	143.6 ± 215.0.	184.5 ± 288.8	1.28	<0.001
TLG_35%_	132.7 ± 179.5	145.2 ± 206.5	1.09	<0.05
TLG_40%_	112.8 ± 148.3	125.8 ± 173.3	1.12	<0.01
TLG_45%_	96.2 ± 124.7	108.1 ± 145.3	1.12	<0.01
TLG_ARG_	196.4 ± 290.0	249.4 ± 383.5	1.27	<0.001

## Discussion

In this study of volume-based parameters on FDG-PET for NSCLC, we found high intra- and inter-operator reproducibility for all parameters (ICC >0.99 each). We also evaluated the time sensitivity of the parameters by comparing early-phase images with delayed-phase images. Whereas the SUVmax increased significantly at the delayed phase, the MTV changes depended on the delineation method, and the TLG obtained by any of the delineation methods was significantly increased at the delayed phase (*P* < 0.05). Among the parameters examined, only MTV_2.0_ and MTV_2.5_ were the parameters that neither increased nor decreased at the delayed phase.

### Intra- and inter-operator reproducibility

In case that the tumor exists without adjacent non-tumor uptakes (i.e., physiological or inflammatory), the semi-automated methods we employed in this study should not cause variability of measurement theoretically. However, it is not uncommon that the tumor is so close to mediastinum that the manual ROIs include parts of blood pool or lymph nodes. In such cases, even semi-automated methods are expected to cause some variation if the threshold is lower than the non-tumor uptake. In this study, we observed both the intra- and inter-operator reproducibility were high for all parameters. Although we observed minimal differences in some cases between the two measurements when relatively low threshold (absolute or fixed% SUVmax) was used, as expected, we consider that the high ICCs may allow use of the methods. Shah et al. reported high inter-operator reproducibility of MTV and TLG using a fixed% SUVmax threshold method that showed the ICCs between two measurements by one operator as > 0.98 for MTV and > 0.99 for TLG [33]. Frings et al. demonstrated high repeatability in the same examination of the two measurement within 1 week using FDG or 18F-fluorothymidine (FLT) [34]. Our results are in line with these previous reports. The difference we observed may be small enough for clinical use.

In contrast, using the ARG method, the twice-measurements of the tumor volume completely agreed, because this method delineates the tumor boundary without requiring a manual ROI [26]. Our results are consistent with this report in terms of high inter-operator reproducibility. However, as a shortcoming, this method does not always successfully determine the tumor boundary, especially when images are noisy or the boundaries are indistinct (or ambiguous). Conducting phantom experiments, Li et al. reported that the ARG method generates a slightly larger volume than the actual tumor volume, and that the degree of volume overestimation depended on the source-to-background ratio. They thus recommended that use of the ARG method should be followed by an appropriate volume correction.

### Early and delayed scans

MTV is the volume where the tumor cells are actively metabolizing glucose. Note that MTV is not an uptake quantification. The volume should not change within a few hours but should be stable if there is no significant tumor growth. In fact, however, many methods of MTV measurement resulted in significant volume changes from the early phase to the delayed phase except for MTV_2.0_ and MTV_2.5_. In contrast, TLG is the arbitrary amount of glucose metabolized during the period from injection to image acquisition. Thus, TLG may change over time theoretically. In the present study, we investigated malignant tumors only; thus, the FDG inflow is thought to continue even 1 h after the injection, resulting in higher uptake at 2 h [35, 36]. Among the MTVs measured by different methods, MTV_2.0_ and MTV_2.5_ neither increased nor decreased from the early to the delayed phase, probably because the increase in tumor uptake and the decrease in the surrounding background uptake (e.g., in a lung field or mediastinum blood pool) would have cancelled each other out. Conversely, the MTV_35%_, MTV_40%_, and MTV_45%_ values all significantly decreased because the increase in the SUVmax raised the delineation cut-off value. MTV_ARG_ increased due to the increase in the tumor-to-background ratio at the delayed phase. TLG by all delineation methods significantly increased; this is likely due to the increase in the SUVmean within the region. Our present report is the first to show parameter changes from the early to delayed phases.

PERCIST, the guideline for PET response criteria in solid tumors, requires that a PET scan for baseline should be obtained at 50–70 min after injection, and the follow-up scan should be obtained within 15 min of the baseline scan [13]. In our observation, almost all parameters changed from the early phase to the delayed phase, which further supported the importance of time strictness. However, it is not always easy to perform scanning under such a strict protocol in many clinical conditions. In particular, when we try to carry out a retrospective analysis, the uptake time restriction will exclude a number of scans. We suggest that use of MTV_2.0_ or MTV_2.5_ could be an alternative way to minimize the influence of uptake time variability.

It should be noted that MTV_2.5_ is the most commonly used method thus far, and is known to be well correlated with patient outcomes of various cancers [27, 28, 37]. For instance, Kao et al. showed that MTV_2.5_ was the most appropriate parameter for predicting recurrence after radiotherapy for pharyngeal cancer patients in comparison with MTV_3.0_, MTV_40%_, and MTV_50%_ [28]. Based on our present findings, MTV_3.0_ or MTV with higher thresholds may not be appropriate if the uptake time is not constant. Another reason to avoid higher thresholds is that a significant number of cases showed zero volume using such thresholds.

MTV_q%_has also been frequently used. MTV_q%_ is actually better at tumor volume measurements in a phantom study because it is relatively resistant to partial volume effects. However, this method may appropriately work when the tumor has intermediate SUVmax (e.g., 5–10) but may under- or over-estimate the volume in cases of considerably high or low SUVmax of tumor, respectively. Therefore, it is difficult to fix relative threshold (%) in studies investigating a large number of patients. Considering the difficulty in fixing an absolute or relative SUV threshold, the ARG procedure is an attractive method that does not require manual interaction. Although the ARG method did achieve very good intra- and inter-operator reproducibility in the present study, its high sensitivity to uptake time necessitated further improvement. TLG seems to extract more information of PET than MTV does, because TLG is an uptake quantification whereas MTV is just a volume. Superiority of TLG to MTV for treatment response of lung cancer has been reported recently [38, 39]. As mentioned above, however, the TLG obtained by any of the delineation methods was significantly increased at the delayed phase. Therefore, when we use datasets acquired with a fluctuating uptake time, we recommend that MTV_2.5_ should be chosen as the best volume-based parameter among many MTVs and TLGs.

The limitations of this study include the following. We investigated reproducibility and parameter changes by uptake time, but we did not report the prognostic value. Future studies will be needed to combine the present findings and prognostic information. In addition, it is necessary to study cancers other than lung cancer. For lung cancer, a manual ROI was defined relatively easily because the tumor existed in the lung showing low FDG uptake. Reproducibility may be affected in fields that have higher physiological uptake, such as the head-and-neck and pelvis.

## Conclusions

The MTV and TLG of primary lesions of 50 NSCLC patients were measured with different tumor delineation methods and different uptake times. We found that both the intra- and inter-operator reproducibility were extremely high for all parameters. Most of the MTV values and all of the TLG values were significantly affected by the uptake time. Among the various parameters studied, MTV_2.0_ and MTV_2.5_ were the least sensitive to the uptake time, and may be good alternatives when we compare images acquired with different uptake times, although applying constant uptake time is important for volume measurement.

## Abbreviations

CT, computed tomography; DRAMA, dynamic row-action maximum likelihood algorithm; FDG, fluorodeoxyglucose; FLT, fluorothymidine; ICC, intra-class correlation; ICCop1, ICC between the first versus second measurement by operator-1; ICCop1op2, ICC between operator-1 versus operator-2; MRI, magnetic resonance imaging; MTV, metabolic tumor volume; NSCLC, non-small cell lung cancer; PET, positron emission tomography; RAMLA, row-action maximum likelihood algorithm; ROIs, regions of interest; SUV, standardized uptake value; SUVmax, maximum of SUV; TLG, total lesion glycolysis; VOI, volume-of-interest
